# CDK-Taverna 2.0: migration and enhancements of an open-source pipelining solution

**DOI:** 10.1186/1758-2946-3-S1-P5

**Published:** 2011-04-19

**Authors:** A Truszkowski, S Neumann, A Zielesny, E Willighagen, C Steinbeck

**Affiliations:** 1 Chemoinformatics and Metabolism, European Bioinformatics Institute (EBI), Cambridge, UK; 2 GNWI – Gesellschaft für naturwissenschaftliche Informatik mbH, Oer-Erkenschwick, Germany; 3 University of Applied Sciences of Gelsenkirchen, Institute for Bioinformatics and Chemoinformatics, Recklinghausen, Germany; 4 Department of Pharmaceutical Biosciences, Uppsala University, Uppsala, Sweden

## 

Pipelining or workflow tools allow for the Lego™-like, graphical assembly of I/O modules and algorithms into a complex workflow which can be easily deployed, modified and tested without the hassle of implementing it into a monolithic application.

The CDK-Taverna project aims at building an open-source pipelining solution through combination of different open-source projects such as Taverna [1], the Chemistry Development Kit (CDK) [2,3] or Bioclipse [4]. A first integrated version of CDK-Taverna was recently released to the public [5].

Current developments in CDK-Taverna refactor all workers as well as the complete setup on the basis of Taverna 2.2 and CDK 1.3.5 which themselves introduce major improvements to the whole platform. In addition the CDK is enhanced with specific functions and options for reaction enumeration based on a reaction template and corresponding reactant libraries. Reaction enumeration supports combinatorial chemistry approaches in the drug discovery process of the pharmaceutical industry. The CDK enhancements are applied and illustrated by corresponding CDK-Taverna-workflows.

## References

[B1] Oinn T Addis M Ferris J Marvin D Senger M Greenwood M Carver T Glover K Pocock MR Wipat A Li P Taverna: a tool for the composition and enactment of bioinformatics workflows Bioinformatics 2004 20 17 3045 3054 10.1093/bioinformatics/bth361 15201187

[B2] Steinbeck C Han YQ Kuhn S Horlacher O Luttmann E Willighagen E The Chemistry Development Kit (CDK): An open-source Java library for chemo- and bioinformatics J Chem Inf Comput Sci 2003 43 2 493 500 12653513 10.1021/ci025584y PMC4901983

[B3] Steinbeck C Hoppe C Kuhn S Guha R Willighagen EL Recent Developments of The Chemistry Development Kit (CDK) - An Open-Source Java Library for Chemo- and Bioinformatics Curr Pharm Design 2006 12 17 2111 2120 10.2174/138161206777585274 16796559

[B4] Spjuth O Helmus T Willighagen EL Kuhn S Eklund M Steinbeck C Wikberg JE Bioclipse: An open rich client workbench for chemo- and bioinformatics BMC Bioinformatics 2007 8 59 17316423 10.1186/1471-2105-8-59 PMC1808478

[B5] Kuhn T Willighagen EL Zielesny A Steinbeck S CDK-Taverna, an open workflow environment for cheminformatics BMC Bioinformatics 2010 11 159 10.1186/1471-2105-11-159 20346188 PMC2862046

